# RNA interference, growth and differentiation appear normal in African trypanosomes lacking Tudor staphylococcal nuclease

**DOI:** 10.1016/j.molbiopara.2010.06.006

**Published:** 2010-11

**Authors:** Sam Alsford, Louise E. Kemp, Taemi Kawahara, David Horn

**Affiliations:** London School of Hygiene & Tropical Medicine, Keppel Street, London, WC1E 7HT, UK

**Keywords:** Ribonuclease, RISC, *Trypanosoma brucei*

## Abstract

Ribonucleases play important roles in the RNA interference (RNAi) pathway. The Dicer endonuclease converts double-stranded (ds)RNA into small interfering (si)RNA and the Slicer endonuclease, as a component of the RNA induced silencing complex (RISC), cleaves mRNA. Tudor staphylococcal nuclease (Tudor-SN) is another component of RISC in humans, flies and nematodes and is therefore implicated in the RNAi pathway. Here, we explore the potential role of African trypanosome Tudor-SN in RNAi. First, we assembled *tudor-sn* null mutants and showed that the gene is dispensable for normal growth and for differentiation. Next, we developed an inducible RNAi reporter system and demonstrated that Tudor-SN is dispensable for RNAi. The kinetics of mRNA knock-down, protein knock-down and protein recovery following inactivation of dsRNA expression are all unperturbed in the absence of Tudor-SN. We conclude that if this nuclease plays a role in the destruction or processing of dsRNA, mRNA or siRNA in the RNAi pathway, it is likely a minor one.

## Graphical abstract

Tudor staphylococcal nuclease is a component of the major RNA interference effector complex, RISC. Using a novel reporter system, we show that Tudor-nuclease is dispensable for dsRNA, mRNA or siRNA processing.

Small eukaryotic RNAs play diverse roles in regulating cellular function. A number of ribonucleases (RNases) function in these pathways but our understanding of the mechanisms involved remains incomplete [1]. Much attention has focused on RNA interference (RNAi), a pathway triggered by dsRNA that serves as an innate defence against viruses, transposons and other ‘selfish’ elements [2], as well as in the regulation of endogenous genes. RNAi has also been exploited as a powerful functional genomics tool [3]. The RNAi trigger, dsRNA, is processed into siRNA (21–26 nt) by a Dicer endo-RNase [4]. In some organisms, once siRNA is loaded into the RNA induced silencing complex (RISC), other RNases such as the argonaute protein, RDE-1 [5] and C3PO, a complex of Translin and Trax [6], promote RISC activation by removing the passenger strand from the siRNA duplex. The remaining siRNA guide strand then facilitates sequence-specific mRNA cleavage, mediated by an endo-RNase known as Slicer, which is invariably a member of the argonaute protein family [7]. Meanwhile, an effector degrading RNase, such as ERI-1, which targets siRNA [8], can limit this response.

RNAi does not operate in all trypanosomatids, but in African trypanosomes, two Dicer nucleases, DCL1 and DCL2 [9], and a Slicer nuclease, AGO1 [10,11], function in RNAi; a role in transposon silencing has been demonstrated [12] and RNAi is widely used as a functional genomics tool [13,14]. The functional replacement of AGO1 in *Trypanosoma brucei* with human Ago2 indicates that RISC structure and function are broadly conserved from trypanosomes to humans [15].

Dicer and Slicer are the only RNases currently known to be essential for RNAi, but additional RNases can clearly impact on the RNAi pathway by degrading dsRNA, mRNA or siRNA. It is interesting in this regard that the RISC complex in humans, flies and nematodes incorporates a phylogenetically conserved Tudor staphylococcal nuclease (Tudor-SN, aka p100 and SND1) of unknown function [16]. In *Tetrahymena*, Tudor-SN appears to play a role in RNA-mediated, programmed DNA rearrangement and is not required for RNAi [17]. The protein has also been implicated in transcription and splicing control [18] and the assembly of Tudor-SN into RISC may antagonise RNAi by functioning in the cleavage of hyper-edited dsRNA in animals [19,20]. Thus, a role for Tudor-SN in the processing of RNAi effectors or products remains unclear.

We explored the potential role of Tudor-SN in the RNAi pathway in the African trypanosome. A regulated reporter system was developed for monitoring the kinetics of mRNA and protein knockdown and the consequences of inactivating dsRNA expression. Using this system, we showed that trypanosome Tudor-SN is dispensable for effective mRNA degradation. In addition, the RNAi effect was reversed with similar kinetics in wild-type Tudor-SN and null *tudor-sn* trypanosomes suggesting that the stability of dsRNA and siRNA effectors was unperturbed in these cells.

The *T. brucei Tudor-SN* gene (GeneID: Tb11.01.5780) encodes a protein with a predicted molecular mass of 100.5 kDa. The protein contains four tandem repeats of a staphylococcal nuclease-like domain (SN1–SN4 in Fig. 1A) and a *C*-terminal Tudor domain. The latter domain is thought to bind proteins containing symmetrically dimethylated arginines, which may be the basis of assembly into effector complexes [18]. To investigate the function of *T. brucei* Tudor-SN, we constructed null mutant strains in bloodstream-form trypanosomes (Fig. 1B). The growth rate of these *tudor-sn* cells and differentiation to the insect-stage in culture was indistinguishable from wild-type (data not shown) indicating that the gene is dispensable for differentiation and growth in both of these life-cycle stages. The Dicer [9] and Slicer [10,11] endo-RNases are dispensable for growth in *T. brucei* so this outcome is not inconsistent with a role in RNAi.

We next set out to explore the potential role of Tudor-SN in the RNAi pathway in *T. brucei*. For this purpose, we constructed a Tetracycline-regulated hairpin expression construct with an integrated, constitutively transcribed, GFP-tagged reporter (Fig. 1C). This ^i^SL^c^GFP system allows for inducible expression of dsRNA and parallel monitoring of GFP reporter expression and also takes advantage of single locus integration for robust and reproducible expression in *T. brucei* [21]. Importantly, preliminary assays using this system indicated that endogenous transcripts and recombinant transcripts were both equally targeted for destruction by the cognate dsRNA (data not shown). To allow for RNAi monitoring in this case, the *T. brucei* gene selected for analysis (GeneID: Tb.11.02.3650) had no discernible effect on growth when disrupted or over-expressed (data not shown). Expression from this and other similar constructs in trypanosomes was substantially higher than from the native alleles (data not shown). This is because the system incorporates a strong RNA polymerase I promoter to drive reporter expression (Fig. 1C) and this facilitates monitoring of the RNAi response without the need for signal amplification.

We then deleted the *Tudor-SN* gene from the ^i^SL^c^GFP reporter strain and selected two null mutants for RNAi analysis. Gene knockout was confirmed in both *tudor-sn* strains by Southern blotting (data not shown, see Fig. 1B). We reasoned that perturbed steps in the RNAi pathway would impact the steady-state levels of mRNA reporter and hairpin RNA species during an induction time-course. In wild-type cells, full-length, GFP-reporter mRNA transcripts were knocked down by ∼85% only 4 h after Tet-addition and ablated 24 h after Tet addition (Fig. 2A). By monitoring expression during this time-frame, we saw no difference in kinetics between wild-type and *tudor-sn* null strains (Fig. 2A, upper panel). A probe for the native-derived portion of the transcript gave similar results, and lower molecular weight products representing hairpin RNA products displayed similar patterns and kinetics in all strains tested (Fig. 2A, lower panel); these latter products were not seen when using a Tb.11.02.3650 probe outside the hairpin fragment (data not shown). These results indicate that Tudor-SN is not required for dsRNA-mediated mRNA degradation in trypanosomes. This nuclease is unlikely to be required for generating the siRNA guide strand within RISC and we can also surmise that Tudor-SN does not substantially antagonise the RNAi pathway in trypanosomes.

We next asked whether Tudor-SN might be involved in inactivating the RNAi response by degrading effectors such as the dsRNA and/or the siRNA guide strand; such roles might not be revealed through monitoring the kinetics of mRNA knockdown. We reasoned that perturbed effector degradation would prolong the recovery of the GFP-reporter after dsRNA expression was interrupted. In this case, we used protein expression as a measure of the RNAi response, which allowed us to assess the combined effect of mRNA degradation and any direct translation interference. As expected, GFP protein expression mirrored mRNA expression and was ablated 24 h after Tet addition (Fig. 2B). The subsequent reversal of RNAi revealed no difference in protein reporter expression kinetics among wild-type and *tudor-sn* null strains (Fig. 2B). Taken together, our results indicate that Tudor-SN does not play a major role in RNAi effector degradation.

The kinetics of mRNA and protein knockdown and recovery were unperturbed in the absence of Tudor-SN in trypanosomes. We conclude that if this nuclease plays a role in the destruction or processing of dsRNA, mRNA or siRNA in the RNAi pathway, it is likely a minor one. The *T. brucei* Tudor-SN gene is syntenic with Tudor-SN homologues in *T. cruzi* and *Leishmania* species so the protein may play a conserved role in trypanosomatids. Future research could address possible roles in transcription, splicing or genomic rearrangement. Beyond Tudor-SN, the reporter system developed for this study represents a useful tool for assessing the function of other putative components and regulators of the RNAi pathway in *T. brucei*.

## Figures and Tables

**Fig. 1:**
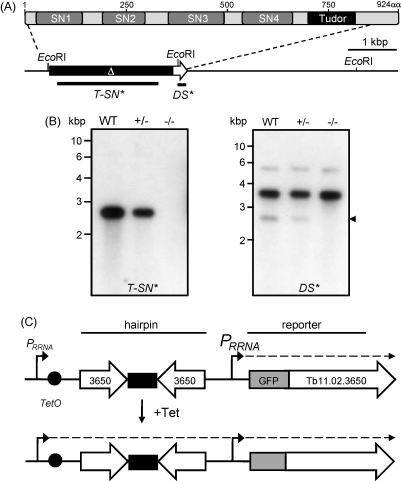
Tudor-SN knockout and an assay system for RNAi regulators. (A) Schematic map of the *T. brucei* Tudor-SN protein and genomic locus. The staphylococcal nuclease (SN1–4) and Tudor domains are indicated. The genomic map illustrates the Tb11.01.5780 locus, the region deleted (Δ) and the probes used for Southern blotting in B. For gene knockout, targeting fragments were amplified from the 284h01 genomic clone. The native alleles (all four SN domains and the tudor domain) were replaced with blasticidin S deaminase (*BSD*) and puromycin *N*-acetyltransferase (*PAC*) selectable markers. We used *T. brucei* Lister 427 bloodstream form MITat1.2 (clone 221a) for all analyses. The bloodstream form cells were grown in HMI-11, transformed with linear DNA constructs and differentiated to the insect stage in DTM as described [21]. (B) Southern blot indicating generation of *tudor-sn* heterozygotes and null strains in bloodstream form cells. Genomic DNA was digested with EcoRI and the blot was sequentially hybridised with the probes indicated. The arrowhead indicates residual signal from the T-SN* probe. Southern analysis was carried out according to standard protocols. (C) Schematic map of the pRPa^iSL-GFPx^ construct which constitutes the ^i^SL^c^GFP assay system for RNAi regulators. pRPa^iSL-GFPx^ was derived from pRPa^iSL^[21]. The long hairpin comprises inverted 519 bp Tb.11.02.3650 fragments. *P*_*RRNA*_, *rRNA* promoter; *TetO*, Tet operator. Genes and gene fragments were amplified by PCR from genomic DNA or plasmid clones using Taq or Fusion high fidelity DNA polymerase (NEB). All oligonucleotide sequences are available upon request.

**Fig. 2:**
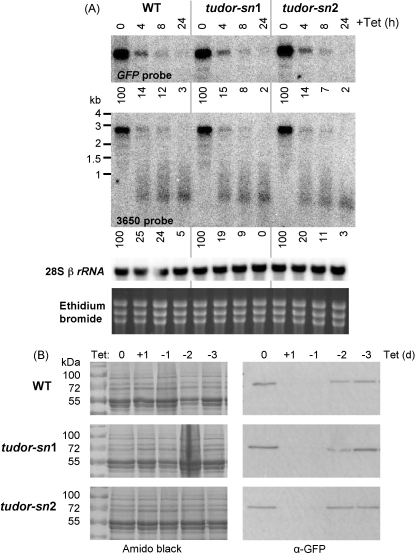
Trypanosome Tudor-SN is not required for RNAi. (A) Northern blots showing RNAi knockdown of ^GFP^3650 during 24 h RNAi induction (+Tet at 1 μg ml^−1^). Tudor-SN was knocked out in 2T1 cells [20] with the ^i^SL^c^GFP reporter system. Northern blots were hybridised with a full length *GFP* probe, a Tb.11.02.3650 probe (fragment used in the hairpin) and a *28Sβ rRNA* probe. The ethidium bromide stained gel is also shown. Northern blotting was carried out according to standard protocols and full-length mRNA signals were quantified using a phosphorimager (Amersham); relative values shown below each image. (B) Western blots showing loss of the ^GFP^3650 protein following 24 h RNAi induction, and recovery after Tet removal; cells were washed in medium lacking Tet and transferred to fresh growth medium. Whole cell lysates were separated by SDS-PAGE and electroblotted using standard protocols. Blots were stained with amido black and probed with a α-GFP rabbit polyclonal sera (Molecular Probes) and goat anti-rabbit HRP conjugated antibody (BioRad). Signals were detected using an ECL + Kit (Amersham) according to the manufacturer's instructions.
